# DCSFormer: a high-precision method for cotton seedling point cloud organ segmentation

**DOI:** 10.3389/fpls.2025.1724451

**Published:** 2026-01-22

**Authors:** Tengfei Liu, Weili Sun, Haoyu Jiang, Luxu Tian, Chenhao Jin, Chenghao Wang, Jicheng Cao, Cairong Chen, Fei Hu

**Affiliations:** 1 College of Smart Intelligence (College of Artificial Intelligence), Nanjing Agricultural University, Nanjing, China; 2 College of Engineering, Nanjing Agricultural University, Nanjing, China

**Keywords:** CLFSkip, cotton point cloud, DCS Block, DCSFormer, organ segmentation

## Abstract

**Introduction:**

Accurately segmenting cotton seedling organs from 3D point clouds is fundamental for high-throughput plant phenotyping and digital breeding. However, cotton seedling segmentation remains challenging due to fine-scale and complex organ morphology, uneven point density with noise, and the lack of high-quality annotated datasets.

**Methods:**

To address these issues, we propose DCSFormer, a tailored extension of Point Transformer V3 designed for cotton seedling point cloud segmentation. The model introduces the DCS Block, which leverages dynamic sparse expert routing and dual-channel attention to adaptively capture global semantic dependencies and subtle local geometric variations, thereby improving stem-leaf boundary discrimination. In addition, the proposed CLFSkip replaces traditional skip connections with a cross-layer fusion strategy, effectively integrating multi-scale features while preserving organ-level details. We also constructed an annotated cotton seedling dataset to support training and evaluation.

**Results and Discussion:**

Experimental results show that DCSFormer achieves 93.67% mIoU, 95.83% mPrec, 97.35% mRec, and 96.56% mF1, outperforming multiple comparison models. Furthermore, when evaluated against baseline models on two public datasets, Crops3D and Pheno4D, DCSFormer exceeds the baseline across all four metrics, further validating its effectiveness and generalizability. This work provides an effective solution for precise cotton seedling organ segmentation.

## 1 Introduction

Cotton is one of the most important economic crops globally, with its fibers widely used in the textile industry, playing a key role in promoting agricultural economic development (Johri et al., 2024). With the continuous advancement of agricultural intelligence and breeding technologies, high-throughput acquisition and analysis of crop phenotypes have become a crucial focus in modern agricultural research (Tietze et al., 2025; Zhao et al., 2025). As an important carrier of phenotypic information, the accurate acquisition of plant organs is critical for understanding crop growth status and assisting breeding decisions (Zhao et al., 2019). Achieving precise segmentation of cotton seedling organs enables the extraction of important plant growth metrics, such as leaf area, leaf curling, volume, and plant height, which are essential for breeding, disease diagnosis, and growth monitoring (Chu et al., 2025). Moreover, effective organ segmentation provides a foundation for accurate measurement of crop phenotypic parameters and helps agricultural practitioners design effective crop management strategies. Therefore, efficient and accurate organ segmentation can enhance cotton production efficiency and promote the development of digital breeding.

Significant progress has been made in plant organ segmentation research. The field has evolved from early manual segmentation methods to modern deep learning-based approaches, reflecting a major transformation in plant organ segmentation (Lei et al., 2024). The development of neural network architectures such as CNNs, RNNs, GNNs, and Transformers (Kim, 2014; Elman, 1990; Scarselli et al., 2008; Vaswani et al., 2017) has advanced deep learning-based segmentation methods. For example, Minervini et al. (2015) employed CNNs to achieve efficient and accurate 2D plant organ segmentation. However, 2D images have inherent limitations, as they cannot fully capture the 3D structure of plants (Ye et al., 2023), making it difficult to segment complex plant structures in 2D imagery.

To address the limitations of 2D image-based segmentation, 3D point clouds have attracted increasing attention due to their ability to provide additional texture and structural information, allowing the extraction of more complex and diverse features (Guo et al., 2020). With the introduction of the PointNet network (Qi et al., 2017b), it became possible to process irregular, unordered point cloud data directly without converting it into voxels or projecting it into 2D images, preserving the integrity of the original 3D information. Subsequent work, PointNet++, introduced a hierarchical feature extraction mechanism (Qi et al., 2017a), which samples local neighborhoods and aggregates features in a progressive manner, significantly enhancing local detail perception. This provides a more accurate 3D representation for plant organ segmentation, advancing point cloud-based phenotyping research.

Building on these networks, Jiang et al. (2025) combined PointNet++ with inverted residual multilayer perceptrons to achieve efficient and accurate apple tree organ segmentation. Song et al. (2025) proposed CotSegNet, which integrates PointNet++ with attention mechanisms to efficiently extract and combine multi-scale features, enhancing segmentation of cotton point cloud data. Beyond PointNet-based methods, Wang et al. (2019) used GNNs to improve segmentation accuracy of plant point clouds in complex backgrounds. Gong et al. (2021) proposed Panicle-3D, which effectively addressed rice panicle point cloud segmentation. Similar advancements have been achieved in maize, soybean, and other crops (Miao et al., 2021; Zhou et al., 2019).

The development of point cloud-based plant organ segmentation techniques provides important reference and inspiration for cotton seedling organ segmentation. However, significant challenges remain. First, cotton seedlings have small and structurally complex organs at early stages (Rehman and Farooq, 2019), making it difficult for traditional methods to capture fine-grained point cloud features, which affects organ-level segmentation accuracy. Second, although cotton seedling point clouds retain spatial geometric information well, uneven point density, noise, and environmental complexity introduce redundant and complex features, negatively impacting segmentation accuracy. Moreover, unlike other domains with established general datasets, high-quality, accurately annotated cotton seedling point cloud datasets are currently lacking, which significantly constrains model training and evaluation and limits further performance improvement.

Based on existing methods and their limitations, this study introduces DCSFormer, a tailored extension of Point Transformer V3 designed for cotton seedling organ point cloud segmentation. The model employs the proposed DCS Block (Dynamic Sparse Attention + Channel + Spatial Attention Mechanism) combined with multi-expert sparse routing, which enhances global features while paying focused attention to fine details, fully exploiting point cloud features and capturing small-scale organ characteristics. Unlike traditional skip connections, DCSFormer introduces CLFSkip (Cross-Layer Fusion Skip), a cross-layer fusion module that extracts and integrates multi-scale features from different encoding layers, enabling the network to comprehensively understand feature information. Additionally, a frequency-domain filter is incorporated to adjust the weights of features at different frequencies, emphasizing or suppressing information to further improve segmentation performance. Overall, we introduce a tailored extension of Point Transformer V3 (DCSFormer) with mixture-of-expert attention and a frequency-aware cross-layer skip connection, specialized for crop organ point clouds. Finally, a high-quality annotated cotton seedling point cloud dataset was constructed to provide a reliable foundation for training and evaluation in this study and for future related research.

## 2 Materials and methods

### 2.1 Data acquisition

Currently, due to the lack of annotated point cloud datasets specifically for cotton seedlings, this study used the experimental cotton variety “Xinjiang Big Peach Cotton” (Gossypium hirsutum L.) to construct a cotton seedling point cloud dataset for model training purposes.

The steps for point cloud data acquisition were as follows: (1) Each video was recorded using a smartphone equipped with a high-definition RGB camera (detailed parameters are listed in **Table 1**). During acquisition, the smartphone was mounted on a fixed-height stabilizing stand and moved around each plant at a uniform speed, ensuring a consistent radius and avoiding abrupt motion. The seedlings were placed in an indoor environment without airflow disturbance, and uniform diffuse LED illumination was used to minimize shadows and photometric variation. (2) An automated Python script was used to extract images from the recorded videos at a rate of one frame every two frames. (3) High-quality, noise-free images were manually selected for point cloud generation. (4) Point cloud data were generated using the software Agisoft.

**Table 1:** Smartphone camera module specifications.

Parameter	Specification
Model	Sony IMX707
Resolution (MP)	50
Aperture	f/1.9
Video Resolution (K)	4
Video Frame Rate (fps)	60

### 2.2 Data preprocessing

Considering the limitations of the environment and equipment, the collected point cloud data inevitably contained background points and noise. To eliminate the impact of these irrelevant points on model evaluation, the data were imported into the 3D point cloud processing software CloudCompare for manual cleaning and annotation. The annotation was performed by two trained annotators following a predefined guideline that specifies the semantic boundaries of each organ type. The guideline was developed in alignment with the annotation criteria used in the Crops3D dataset (Zhu et al., 2024), with minor adaptations to account for the morphological characteristics of cotton seedlings. It includes detailed rules for handling ambiguous regions such as stem–leaf junctions, occluded leaves, and partially deformed structures. To ensure consistency, approximately 20% of the samples were cross-checked by both annotators, and any disagreements were resolved through discussion to establish a unified labeling standard.

During preprocessing, noisy background points were first removed manually, followed by statistical outlier filtering to eliminate residual noise and ensure high-quality point cloud data for subsequent segmentation. After cleaning, the point clouds were semantically classified into organ categories according to the labeling scheme presented in **Table 2**. Each sample was annotated with semantic labels, and instance labels were also generated but not used in this study; these instance-level annotations may support future research on instance segmentation, fine-grained organ morphology, or detailed phenotyping tasks.

**Table 2:** Point cloud label mapping.

Class	Semantic label	Instance label	Description
Leaf	1	1-N	Each leaf has a unique instance ID
Stem	2	1-N	Each stem segment has a unique instance ID
Pot (non-plant point)	0	0	Pot and other non-plant points, no instance differentiation

In addition, basic ground-truth phenotypic traits such as leaf count, plant height, and canopy width were recorded during the annotation process. Leaf count was used to verify annotation completeness, and plant height and canopy width served as auxiliary quantitative indicators for segmentation evaluation.

After data processing, 100 initial point cloud datasets were generated. To avoid model overfitting and ensure that the model can effectively process feature information, the point cloud data were split into training and testing sets at a 4:1 ratio. All data augmentation operations were applied exclusively to the training set. The 80 training samples were expanded to 720 samples through random rotation, Gaussian noise addition, random point removal, and jittering. The test set consisted solely of the original, non-augmented samples to prevent any risk of data leakage.

### 2.3 DCSFormer

**Figure 1:**
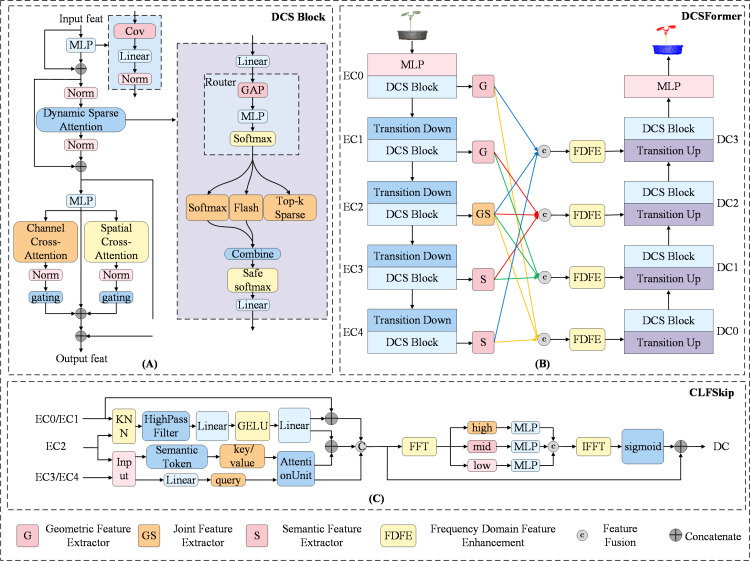
Overview of the proposed DCSFormer. **(A)** The DCSBlock combining dynamic sparse attention, channel attention, and spatial attention. **(B)** The overall architecture of DCSFormer. **(C)** CLFSkip module for cross-layer feature fusion via skip connections.

In this study, the DCSFormer model is proposed for organ-level segmentation of cotton seedling point clouds. The model architecture is developed based on Point Transformer V3 (Wu et al., 2024). As shown in **Figure 1B**, the overall structure of DCSFormer adopts an encoder–decoder framework. Compared with the baseline model, a more task-suitable DCS Block is introduced to replace the original block in the baseline.

Within the DCS Block, a multi-expert sparse routing mechanism dynamically selects a small subset of the most relevant experts according to the input features. During inference, different experts can focus on distinct geometric or semantic patterns, thereby enhancing the model’s adaptability to diverse point cloud structures. Meanwhile, the dual-channel attention mechanism balances local details with global dependencies, improving the selectivity and discriminability of feature representations while reducing interference from irrelevant information. As a result, the DCS Block provides stronger generalization and robustness for complex organ-level plant point cloud segmentation, enabling the model to better capture subtle differences between leaves and stems, thus improving segmentation accuracy.

For the critical skip-connection component in the encoder–decoder framework, a novel CLFSkip design is proposed to replace the classic skip connections used in U-Net. CLFSkip optimizes the connections according to the feature properties of different encoder layers, and subsequently integrates features across layers with varying resolutions and semantic levels. This design balances fine-grained details with contextual information, enriching the decoder’s input with multi-scale and gradient information.

#### 2.3.1 DCS block

The multi-head attention mechanism designed in Point Transformer V3 has demonstrated strong performance in point cloud processing, as it prioritizes key features, suppresses less relevant ones, and enables efficient feature interaction and representation. However, in the task of organ-level segmentation of cotton seedlings, challenges arise due to the complex structure of leaves, frequent occlusions, and easily confused boundary regions between overlapping leaves. Moreover, the slender stems differ significantly from leaves in both geometric and semantic space (Muraleedharan and Rajan, 2024). Although traditional multi-head attention can dynamically adjust the importance of points, it struggles to highlight subtle differences along leaf edges and adapt to diverse local geometric and semantic features. Consequently, in scenarios with blurred organ boundaries or large morphological variations, its limitations can lead to reduced segmentation accuracy.

To address these shortcomings, the DCS Block is proposed to replace the original block, specifically targeting the limitations of conventional attention in emphasizing leaf edge details and capturing stem–leaf relationships. As illustrated in **Figure 1A**, the DCS Block introduces a sparse router that dynamically selects two of the most suitable experts based on the global features of each local patch, rather than relying on a fixed attention computation scheme. Different experts correspond to different types of attention, enabling the model to adaptively align with diverse local structural features. A balanced loss is applied to mitigate routing bias and ensure fair utilization of experts, thereby enhancing the model’s adaptability to varied geometric and semantic contexts.

For this study, the dynamic sparse attention framework employs three types of attention mechanisms as experts: Softmax Attention, Flash Attention, and Top-k Sparse Attention. Softmax Attention establishes comprehensive global dependencies across the point cloud, ensuring effective interaction between organs such as leaves and stems. Flash Attention provides efficient global modeling for long-sequence point clouds, avoiding memory and computational bottlenecks. Top-k Sparse Attention focuses on key local neighborhoods, which is particularly beneficial for accurately capturing fine-grained details along leaf edges and stem regions. In the DCS Block, we use H heads per expert, with all experts sharing the same Q/K/V dimensions (d). The Q/K/V projections are shared across all experts to enhance model efficiency. Each expert operates on the same set of patch tokens, and Top-k Sparse Attention selects k neighbors per query token based on dot-product similarity, allowing the model to focus on the most relevant tokens while maintaining computational efficiency. Collectively, these three experts complement one another, balancing accuracy and efficiency, and ultimately improving the precision and effectiveness of cotton seedling organ segmentation.

In addition, this component leverages an MLP with local convolution to implicitly encode the relative positional information of points. The points are divided into patches, with each patch consisting of 512 points. The local convolution is applied within a neighborhood of points defined by KNN (k=16), where each point’s neighborhood consists of its 16 nearest neighbors. The convolution is performed using a SubMConv3d layer with a kernel size of 3, which captures local spatial relationships. After the convolution, the encoded information is passed through a Linear layer followed by Norm to further process the features. These processed features are then passed into the dynamic sparse attention and expert routing module. The process can be formally expressed as follows, as shown in Equations 1–8:

Let the token representation of a single input patch be denoted as $X\in {\mathbb{R}}^{N\times C}$, where N is the number of points in the patch and C is the feature dimension. Its global average pooling (GAP) can be expressed as:

(1) $$\begin{array}{c} g=\frac{1}{N}\sum_{i=1}^{N}x_{i} \end{array}$$

The router computes the expert scores through a two-layer MLP, which can be formulated as:

(2) $$\begin{array}{c} z=W_{2}\phi (W_{1}g+b_{1})+b_{2},\;\;z\in {\mathbb{R}}^{E} \end{array}$$

Here, E denotes the total number of experts and ϕ is the activation function. The top-k expert index set is selected as T=TopK(z,k), and a sparse softmax is applied over this set to obtain the sparse gating weights. The temperature parameter τ controls the sharpness of the softmax distribution: a higher τ results in a more uniform distribution, while a lower τ focuses the gating weights on fewer experts, leading to sparser activations:

(3) $$\begin{array}{c} \alpha_{e}=\{\begin{array}{c} \frac{exp(\frac{z_{e}}{\tau })}{\sum_{j\in T}exp(\frac{z_{j}}{\tau })},\;\;\;e\in T,\\ 0,\;\;\;\;\;\;\;\;\;\;e\notin T, \end{array} \end{array}$$

Let the attention mapping of each expert be denoted as $f_{e}(\cdot )$. The attention output of each patch Y is then computed as:

(4) $$\begin{array}{c} Y=\sum_{e=1}^{E}\alpha_{e}f_{e}(X)=\sum_{e\in T}\alpha_{e}f_{e}(X), \end{array}$$

To ensure balanced utilization of experts, the balancing loss $L_{bal}$ is defined as:

(5) $$\begin{array}{c} L_{bal}=\|u-\frac{1}{E}1{\|}_{2}^{2}+\|p-\frac{1}{E}1{\|}_{2}^{2}, \end{array}$$

Here, $u={\left[u_{1},\ldots ,u_{E}\right]}^{T}$ with $u_{i}$ denoting the utilization rate of the i-th expert, $p={\left[p_{1},\ldots ,p_{E}\right]}^{T}$ with $p_{i}$ representing the average weight assigned to the i-th expert, and **1** is an all-ones vector. The total loss is then given by:

(6) $$\begin{array}{c} L=L_{bal}+L_{task} \end{array}$$

Where $L_{task}$ denotes the segmentation task loss. By leveraging dynamic sparse attention and expert routing, the model can adaptively select the most suitable attention strategy for different local geometric patterns, thereby enhancing its feature representation capability.

In addition, the DCS Block integrates CAM (Channel Attention Mechanism, proposed by Hu et al., 2018) and SAM (Spatial Attention Mechanism, proposed by Jaderberg et al., 2015), which respectively enhance inter-channel dependencies and spatial geometric sensitivity. A learnable gating parameter is introduced to adaptively fuse the two mechanisms, thereby reinforcing the model’s response to key structures from both the feature-channel and spatial-distribution perspectives. Consequently, the DCS Block not only retains the advantages of global modeling but also provides fine-grained control over details. The final feature output $x_{out}$ is given as:

(7) $$\begin{array}{c} x_{out}=x+sigmoid(\alpha )\cdot x_{cca}+(1-sigmoid(\alpha ))\cdot x_{sca} \end{array}$$

Here, x denotes the input feature, $x_{cca}$ the output feature from channel attention, and $x_{sca}$ the output feature from spatial attention. A learnable gating parameter sigmoid(α) is introduced to balance the contributions of CCA and SCA, defined as:

(8) $$\begin{array}{c} sigmoid(\alpha )=\frac{1}{1\;+\;e^{-\alpha }} \end{array}$$

Here, α is a learnable gating scalar.

#### 2.3.2 CLFSkip

In point cloud segmentation tasks, spatial domain information is crucial for segmentation accuracy (Zhang et al., 2019). However, the encoder typically involves multiple downsampling operations intended to enlarge the receptive field and capture high-level semantic information. During this process, the resolution of feature maps gradually decreases, leading to a loss of spatial details, which can hinder accurate boundary reconstruction. U-Net–style models (Ronneberger et al., 2015) commonly use skip connections to directly pass shallow encoder features to the corresponding decoder layers, helping the decoder reconstruct features and partially mitigating this issue.

Nevertheless, traditional skip connections exhibit certain limitations in cotton seedling organ point cloud segmentation. First, the junctions between leaves and stems are geometrically similar but semantically distinct. Directly concatenating features can lead to feature-space mismatch and class confusion. Moreover, cotton seedling organs contain numerous thin petioles and delicate leaves, which are prone to losing detail during downsampling. In traditional skip connections, shallow features lack targeted enhancement, making it difficult for the decoder to restore clear boundaries. Additionally, cotton seedling point clouds are often dense in leaf regions but sparse around stems and branches. Directly fusing features from different levels can introduce noise and other interference, reducing robustness in organ classification. Given the high demand for local detail in this task, traditional skip connections, which do not selectively extract features at different frequencies, fail to adequately capture both global structure and local details.

To address these limitations, this study proposes CLFSkip, a skip-connection mechanism with multi-dimensional feature extraction and cross-layer fusion. During the skip-connection stage, it introduces a differential feature extraction enhancement module and cross-layer frequency fusion to achieve more effective feature transmission and utilization. The module structure is illustrated in **Figure 1C**, and the corresponding formulation is given in Equations 9–19.

During encoder downsampling, features progressively transition from local geometric textures to more abstract semantic representations (Gu et al., 2017). Accordingly, CLFSkip implements a layer-specific feature extraction enhancement strategy: for shallow features (Enc0/Enc1), which primarily contain spatial structures and boundary information, local details are further emphasized by constructing high-pass residuals via k-NN neighborhoods. For a given point i with feature $x_{i}$ and its k nearest neighbors obtained via k-NN, denoted as χ(i), the neighborhood mean is computed as:

(9) $$\begin{array}{c} {\bar{x}}_{i}=\frac{1}{k}\sum_{j\in \chi (i)}x_{i} \end{array}$$

The constructed high-frequency component, representing the difference between a point and its neighborhood, is defined as:

(10) $$\begin{array}{c} h_{i}=x_{i}-{\bar{x}}_{i} \end{array}$$

which is then passed through an MLP for nonlinear mapping and fused back via a residual connection:

(11) $$\begin{array}{c} x_{i}^{'}=x_{i}+MLP(h_{i}) \end{array}$$

For deep-layer features (Enc3/Enc4), global context modeling is applied to enhance semantic consistency. First, the global semantic token of each block is computed as the mean of all point features:

(12) $$\begin{array}{c} s=\frac{1}{N}\sum_{I=1}^{N}x_{i} \end{array}$$

Where $x_{i}$ is the feature vector of the i-th point, and s is the mean feature of all points in the block (batch). The point features are then projected:

(13) $$\begin{array}{c} q_{i}=W_{q}x_{i} \end{array}$$

and the semantic token is projected as key and value:

(14) $$\begin{array}{c} k=W_{k}s \end{array}$$

(15) $$\begin{array}{c} v=W_{v}s \end{array}$$

where $W_{q}$、 $W_{k}$ and $W_{v}$ are learnable linear transformation matrices. The attention weight of each point to the global token is computed as:

(16) $$\begin{array}{c} \alpha_{i}=Softmax(q_{i}\cdot k) \end{array}$$

and the final output feature is given by:

(17) $$\begin{array}{c} x_{i}^{'}=W_{o}(\alpha_{i}v) \end{array}$$

Where $W_{o}$ is a learnable output projection matrix.

For mid-layer features (Enc2), the enhanced geometric features ( $x_{i}^{geo}$) and semantic features ( $x_{i}^{sem}$) are fused via a gating network to obtain adaptive weights:

(18) $$\begin{array}{c} \alpha_{i}=\sigma (W_{f}[x_{i}^{geo}][x_{i}^{sem}]) \end{array}$$

Where σ denotes the sigmoid function, [·][·] represents concatenation, and $W_{f}$ is the fusion weight matrix. The final combined feature is:

(19) $$\begin{array}{c} x_{i}^{'}=W_{g}(\alpha_{i}x_{i}^{geo}+(1-\alpha_{i})x_{i}^{sem}) \end{array}$$

Where $W_{g}$ is the output projection matrix. This mechanism adaptively balances local geometric details with global semantic information, helping the model distinguish morphologically similar yet semantically different organs in complex plant point clouds.

Finally, cross-layer feature fusion is applied to integrate multi-level semantic and geometric information. The correspondence of cross-layer features is summarized in **Table 3**.

**Table 3:** Cross-layer feature fusion correspondence relationship.

Decoder stage	Fused encoder layers	Output channels
Dec0	Enc0 + Enc2 + Enc4	64
Dec1	Enc1 + Enc2 + Enc3	128
Dec2	Enc0 + Enc2 + Enc4	256
Dec3	Enc1 + Enc2 + Enc3	512

The decoder Dec0 fuses shallow features from Enc0, mid-level features from Enc2, and deep features from Enc4. This design integrates high-resolution geometric details, local structural semantic information, and global contextual semantic features, thereby enabling accurate organ boundary reconstruction while maintaining overall semantic consistency. Similarly, Dec1 fuses features from Enc1, Enc2, and Enc3, primarily focusing on mid-to-high-level features to balance detail and semantic information, supporting the discrimination of complex organ classes. The fusion patterns of Dec0 and Dec2, as well as Dec1 and Dec3, are consistent. This design strategy strikes a balance in feature richness at different levels, while maintaining semantic consistency and computational efficiency. After concatenation, the input channels of the cross-layer fusion are adjusted via linear mapping and nonlinear projection to a unified output channel, ensuring feature dimension consistency and effective information integration.

Since spatial-domain methods often lack sufficient modeling of high-frequency details, CLFSkip introduces a frequency-domain enhancement module to complement global and local features, improving performance in complex structural and fine-detail recognition tasks. Although raw point cloud data are irregular in Euclidean space, the feature maps extracted by the network are represented on a regular tensor grid, making FFT-based frequency decomposition applicable. In CLFSkip, we apply a 1D fast Fourier transform (FFT) along the point dimension of the feature map, treating each channel as a signal defined over points. Within this formulatio, different frequency components correspond to different levels of geometric variation: the low-frequency components (first 1/4 of the spectrum) encode coarse global morphology such as overall plant shape and the arrangement of major organs; the mid-frequency components (middle 1/2) capture medium-scale curvature changes including leaf bending and petiole orientation; and the high-frequency components (final 1/4) represent sharp structural transitions and local discontinuities, which are particularly critical for accurately delineating the stem–leaf boundary. After frequency decomposition, each band is processed by a dedicated multi-layer perceptron (MLP) to enhance its scale-specific information. The enhanced spectra are then recombined and transformed back into the spatial domain via inverse FFT. Finally, a Sigmoid gating mechanism is applied to dynamically weight the contributions of different frequency components, emphasizing low-frequency global context and high-frequency boundary details. This frequency-aware fusion helps the model better distinguish subtle geometric transitions around stem–leaf junctions, thereby improving segmentation accuracy in structurally complex regions.

In summary, CLFSkip not only effectively integrates shallow geometric details with deep semantic information but also leverages frequency-domain enhancement to further strengthen the modeling of fine-grained features.

### 2.4 Experimental indicators

#### 2.4.1 Semantic segmentation metrics

In this study, Intersection over Union (IoU), Precision, Recall, F1 score, and their averages were adopted to evaluate the performance of DCSFormer and comparative models on cotton seedling point cloud organ segmentation, as defined in Equations 20–23.

The mean Intersection over Union (mIoU) is calculated as the average IoU across all segmented organ categories, defined as:

(20) $$\begin{array}{c} mIou=\frac{1}{N}\sum_{i=1}^{N}\frac{TP_{i}}{TP_{i}+FP_{i}+FN_{i}} \end{array}$$

where N is the total number of classes, $TP_{i}$ is the number of true positives, $FP_{i}$ is the number of false positives, and $FN_{i}$ is the number of false negatives for class i.

The mean Precision (mPrec) evaluates the accuracy of the segmentation model. It is computed as the average precision across all classes, with higher values indicating fewer segmentation errors:

(21) $$\begin{array}{c} mPrec=\frac{1}{N}\sum_{i=1}^{N}\frac{TP_{i}}{TP_{i}+FP_{i}} \end{array}$$

The mean Recall (mRec) measures the completeness of the model by averaging the recall over all classes, reflecting the coverage of each category:

(22) $$\begin{array}{c} mRec=\frac{1}{N}\sum_{i=1}^{N}\frac{TP_{i}}{TP_{i}+FN_{i}} \end{array}$$

The mean F1 score (mF1) provides a comprehensive evaluation by combining mean precision and mean recall, indicating whether the model achieves a good balance between accuracy and completeness:

(23) $$\begin{array}{c} mF1=\frac{1}{N}\sum_{i=1}^{N}\frac{2\cdot Prec_{i}\cdot Rec_{i}}{Prec_{i}+Rec_{i}} \end{array}$$

#### 2.4.2 Correlation-based phenotypic evaluation metrics

This study evaluates the effectiveness and suitability of DCSFormer for plant point cloud analysis by comparing algorithmically predicted trait values with manual measurements. The performance was quantified using the coefficient of determination (R²), mean absolute percentage error (MAPE), root mean square error (RMSE), and mean absolute error (MAE), as defined in Equations 24–27.

(24) $$\begin{array}{c} R^{2}=\frac{\sum_{i=1}^{n}(x_{i}-\bar{x})(y_{i}-\bar{y})}{\sqrt{\sum_{i=1}^{n}{\left(x_{i}-\bar{x}\right)}^{2}\sum_{i=1}^{n}{\left(y_{i}-\bar{y}\right)}^{2}}} \end{array}$$

(25) $$\begin{array}{c} MAPE=\frac{1}{n}\sum_{i=1}^{n}\frac{\left|x_{i}-y_{i}\right|}{x_{i}}\times 100\% \end{array}$$

(26) $$\begin{array}{c} RMSE=\sqrt{\frac{1}{n}\sum_{i=1}^{n}{\left(x_{i}-y_{i}\right)}^{2}} \end{array}$$

(27) $$\begin{array}{c} MAE=\frac{1}{n}\sum_{i=1}^{n}|x_{i}-y_{i}| \end{array}$$

where n is the number of plants, $x_{i}$ is the manually measured value for the i-th plant, $\bar{x}$ is the mean of the manual measurements, $y_{i}$ is the algorithm-predicted value for the i-th plant, and ȳ is the mean of the predicted values.

## 3 Results

### 3.1 Experimental environment

All experiments in this study were conducted under the same hardware and software conditions to maximize experimental rigor and ensure fair comparison of results. All experiments were performed on a computing platform running Ubuntu 22.04, equipped with an NVIDIA GeForce RTX 4090 GPU and an Intel(R) Xeon(R) Platinum 8352V CPU @ 2.10GHz. Detailed hardware and software configurations are listed in **Table 4**.

**Table 4:** Hardware and software configuration.

Component	Specification
CPU	Intel(R) Xeon(R) Platinum 8352V CPU @ 2.10GHz
GPU	NVIDIA GeForce RTX 4090
Memory (RAM)	90 GB
Operating System	Ubuntu 22.04
CUDA Toolkit	11.8
Deep Learning Framework	PyTorch 2.1.0
Python Version	3.8

In the ablation experiments, the batch size was set to 2, with gradient accumulation performed over 6 steps (i.e., gradients from 6 batches were accumulated before each parameter update). The learning rate was uniformly set to 0.002. The dataset was split into 5 folds using the S3DIS format, and 5-fold cross-validation was employed for model evaluation, where each fold served as a validation set in turn. This method was used to ensure robust performance evaluation without a dedicated validation set. Hyperparameters, such as the learning rate, were tuned using this cross-validation process, and no early stopping was applied. All other configurations used their default settings.

### 3.2 Ablation experiment

The effectiveness of the proposed DCSFormer model was verified through ablation experiments, with detailed results presented in **Table 5**.

**Table 5:** Ablation experiment results.

Group	Method	mIoU (%)	mPrec (%)	mRec (%)	mF1 (%)
A	Point Transformer V3	90.46	92.82	96.76	94.71
	DCSFormer				
B	— DCS Block	92.74	95.00	97.09	96.02
C	— CLFSkip	92.44	94.53	97.19	95.84
D	— DCS Block+ CLFSkip	**93.67**	**95.83**	**97.35**	**96.56**

Bold type indicates the best result achieved in the corresponding indicator.

During the experiments, a controlled variable approach was used to evaluate the contributions of DCS Block, CLFSkip, and the complete DCSFormer model for cotton seedling organ point cloud segmentation. Comparing Group A and Group B, we observed that the integration of the DCS Block increased mIoU by 2.28% and mF1 by 1.31%, with the largest improvement observed in mPrec, which increased by nearly 3 percentage points. This demonstrates the effectiveness of the DCS Block in enhancing feature representation and improving segmentation accuracy.

When comparing Group A and Group C, the addition of CLFSkip led to a mIoU increase of 1.98% and mPrec increase of 2.37%, indicating that the proposed skip connection design facilitates more effective multi-scale feature fusion and strengthens contextual understanding. Finally, compared to the baseline, Group D—integrating both DCS Block and CLFSkip—achieved the highest performance across all metrics, with mIoU increasing by 3.21%, mPrec by 3.01%, mRec by 0.59%, and mF1 by 1.85%. These results confirm the complementary advantages of DCS Block and CLFSkip and demonstrate that their integration in DCSFormer enhances the overall segmentation capability of the model.

To further evaluate fine-grained segmentation performance, we reported per-class accuracy metrics for each organ category in the cotton seedling point cloud dataset. This analysis provides insight into the robustness of the proposed model in handling different structural components, with detailed results shown in **Table 6**. Comparing the baseline model (Group A) with the B-group model incorporating DCS Block, we observed a significant improvement in stem segmentation, with IoU increasing by 3.67% and Precision by 4.43%, indicating that DCS Block effectively enhances fine-grained feature learning for complex organ structures. The addition of CLFSkip in Group C further optimized the model, particularly for non-plant regions, achieving an IoU of 99.95%. Finally, Group D’s DCSFormer achieved the best overall performance, attaining the highest accuracy across all three organ categories. Specifically, compared to the baseline, stem IoU increased by 5.80%, while leaf and non-plant categories exceeded 98% across all metrics.

**Table 6:** Per-organ segmentation performance comparison.

Group	Method	Organ category	Ins. IoU (%)	Precision (%)	Recall (%)	F1 score (%)
A	Point Transformer V3	Stem	77.14	82.98	91.64	87.09
		Leaf	95.48	96.72	98.68	97.68
		Non-plant	98.76	98.77	99.99	99.37
	DCSFormer					
B	— DCS Block	Stem	80.81	87.41	91.45	89.38
		Leaf	97.62	97.63	**99.99**	98.79
		Non-plant	99.80	99.97	99.83	99.89
C	— CLFSkip	Stem	79.94	86.96	90.83	88.85
		Leaf	97.43	97.65	99.77	98.69
		Non-plant	99.95	98.99	99.99	99.97
D	— DCS Block+ CLFSkip	Stem	**82.94**	**88.22**	**93.26**	**90.67**
		Leaf	**98.11**	**99.29**	98.80	**99.04**
		Non-plant	**99.97**	**99.99**	**99.98**	**99.98**

Bold type indicates the best result achieved in the corresponding indicator.

It is worth noting that, although DCSFormer achieved nearly the best overall performance across all categories, the recall for the leaf class was slightly lower than that of Groups B and C. This phenomenon reflects a trade-off between precision and recall, as DCSFormer prioritizes precision to reduce misclassification, which results in a minor decrease in recall. This outcome indicates that the proposed modules not only improve overall accuracy but also implement a balanced optimization strategy across different categories. In summary, the proposed modules exhibit complementary advantages, and their interaction within the DCSFormer model ensures a balanced performance across different organ classes.

### 3.3 Comparison with state-of-the-art methods

To further investigate the performance of the DCSFormer model in cotton seedling point cloud organ segmentation, several state-of-the-art point cloud segmentation models were selected for comparison under the same experimental conditions. Except for the baseline model, all models were trained according to the default strategies reported in their original papers to achieve the best performance as documented (The specific hyperparameter settings for each model are detailed in the Supplementary Material.). The experimental results are summarized in **Table 7**. In the table, we report four evaluation metrics for each class across all compared methods, as well as the mean and standard deviation over the five-fold cross-validation runs. The observed standard deviations are relatively small (ranging from 0.02 to 0.08), indicating that the results are stable across folds.

**Table 7:** Compare the performance of DCSFormer with SOTA models.

Metrics	Methods	Stem	Leaf	Non-plant	Average	SD
mIoU (%)	PointNet	44.90	88.87	98.73	77.50	0.08
	PointNet++	64.60	88.74	96.43	83.25	0.06
	Point Transformer	73.38	88.61	98.58	86.85	0.06
	PAConv	46.22	90.41	98.97	78.53	0.02
	Point Transformer V3	77.14	95.48	98.76	90.46	0.05
	SPoTr	78.80	93.80	99.08	90.56	0.04
	DCSFormer	**82.94**	**98.11**	**99.97**	**93.67**	0.06
mPrec (%)	PointNet	52.11	98.42	98.10	82.88	0.06
	PointNet++	72.06	92.99	96.83	87.29	0.05
	Point Transformer	80.58	90.27	99.11	89.98	0.03
	PAConv	63.87	88.15	99.92	83.89	0.03
	Point Transformer V3	82.98	96.72	98.77	92.82	0.04
	SPoTr	84.31	98.64	99.75	94.23	0.03
	DCSFormer	**88.22**	**99.29**	**99.99**	**95.83**	0.04
mRec (%)	PointNet	76.43	90.15	**99.99**	89.07	0.09
	PointNet++	86.18	95.10	99.57	93.62	0.06
	Point Transformer	89.14	97.96	99.46	95.52	0.04
	PAConv	62.58	99.04	**99.99**	88.18	0.04
	Point Transformer V3	91.63	98.67	99.98	96.76	0.05
	SPoTr	**93.59**	96.90	99.33	96.60	0.05
	DCSFormer	93.26	**98.80**	99.98	**97.35**	0.08
mF1 (%)	PointNet	61.96	94.10	99.03	85.14	0.07
	PointNet++	78.49	94.03	98.18	90.23	0.06
	Point Transformer	84.64	93.95	99.28	92.63	0.04
	PAConv	63.22	93.27	99.95	85.88	0.02
	Point Transformer V3	87.09	97.68	99.37	94.71	0.04
	SPoTr	88.71	97.76	99.53	95.33	0.05
	DCSFormer	**90.67**	**99.04**	**99.98**	**96.56**	0.06

Bold type indicates the best result achieved in the corresponding indicator.

The quantitative results in **Table 7** demonstrate that DCSFormer outperforms the six other advanced comparison models, achieving the highest scores across all four evaluation metrics. Specifically, PointNet and PointNet++ exhibited clear limitations, with mIoU values of 77.50% and 83.25%, respectively, lagging behind more recent architectures. Although Point Transformer (Zhao et al., 2021) delivered competitive results, its segmentation performance was still surpassed by Point Transformer V3 and SPoTr (Park et al., 2023), which achieved mIoU scores of 90.46% and 90.56%, respectively. In contrast, DCSFormer achieved an mIoU of 93.67%, approximately 3 percentage points higher than both Point Transformer V3 and SPoTr. **Table 8** compares the segmentation efficiency of DCSFormer with six state-of-the-art networks. As shown, DCSFormer incurs higher computational cost than most baselines, exhibiting larger FLOPs as well as longer training and inference times. This indicates that the model is not the most efficient in terms of throughput. However, the increased complexity is consistent with the architectural design, which emphasizes richer geometric representation through dynamic channel–spatial modeling and cross-layer frequency fusion. Consequently, DCSFormer achieves the highest segmentation accuracy among all compared methods—reflecting a deliberate trade-off in which high precision is achieved at the cost of higher computational complexity.

**Table 8:** Comparison of segmentation efficiency using the DCSFormer and six other state-of-the-art networks.

Model	Param (M)	FLOPs (M)	Training time (min)	Inference time (ms)
PointNet	8.3	234	0.12	21
PointNet++	1.7	988	0.42	198
Point Transformer	19.3	1912	26.8	448
PAConv	44.9	1253	0.14	63
Point Transformer V3	46.1	1540	7.1	181
SPoTr	25.7	1233	0.14	65
DCSFormer	49.7	1776	10.4	197

It is worth noting that PAConv (Xu et al., 2021) performed relatively poorly on this task. Neither the relatively stable PyTorch version nor the CUDA-optimized version produced satisfactory results (the PyTorch version data are reported in **Table 7**), with an mIoU of only 78.53%. This suggests that PAConv has certain limitations in capturing global and local features and exhibits slightly weaker global semantic modeling capability for this task.

Regarding the F1 score, DCSFormer also achieved the highest value of 96.56%, outperforming Point Transformer V3 and SPoTr by 1.85% and 1.23%, respectively. This indicates that DCSFormer not only provides strong overall segmentation capability but also maintains a good balance between precision and recall.

From an architectural perspective, DCSFormer integrates global context and local geometric cues more effectively than other models. In contrast, earlier methods such as PointNet and PointNet++ rely on relatively shallow architectures and hierarchical feature grouping, which limits their representational capacity. Even attention-based models, such as SPoTr, fail to fully exploit fine-grained geometric relationships in the context of this study. DCSFormer addresses these limitations by introducing a more powerful feature aggregation strategy, which explains its superior performance across all four metrics.

In summary, for the semantic segmentation of cotton seedling point clouds, DCSFormer achieves state-of-the-art performance, attaining the highest values in mIoU, mPrec, mRec, and F1 score, demonstrating its effectiveness in this application.

### 3.4 Statistical analysis of model performance

To assess the statistical significance of the performance differences among segmentation models, we conducted a one-way analysis of variance (ANOVA) on the mean Intersection over Union (mIoU) across models. The null hypothesis assumes that all models perform equivalently. **Table 9** summarizes the ANOVA results, indicating a statistically significant difference in mIoU among the models (F = 24.8021, p = 1.36e-19).

**Table 9:** Statistical analysis of mIoU among segmentation models.

ANOVA F-statistic	ANOVA p-value	Model1	Model2	Mean difference	Tukey HSD p-value
24.8021	1.36e-19	DCSFormer	PAConv	-0.1513	< 0.0001
		DCSFormer	Point Transformer	-0.0681	0.0045
		DCSFormer	PointNet	-0.1661	< 0.0001
		DCSFormer	PointNet++	-0.1041	< 0.0001
		Point Transformer V3	PAConv	-0.1193	0.0002
		Point Transformer V3	PointNet	-0.1341	< 0.0001
		Point Transformer V3	PointNet++	-0.0721	0.0021

F-statistic and p-value from one-way ANOVA are shown, followed by Tukey HSD *post-hoc* test for model pairs with significant differences (p < 0.05).

To further identify which model pairs exhibit significant differences, we performed Tukey’s Honest Significant Difference (HSD) *post-hoc* test. As shown in **Table 9**, DCSFormer achieves statistically significant improvements in mIoU over PAConv (mean difference = -0.1513, p < 0.0001), Point Transformer (mean difference = -0.0681, p = 0.0045), PointNet (mean difference = -0.1661, p < 0.0001), and PointNet++ (mean difference = -0.1041, p < 0.0001). Similarly, Point Transformer V3 shows significant gains over PAConv (mean difference = -0.1193, p = 0.0002), PointNet (mean difference = -0.1341, p < 0.0001), and PointNet++ (mean difference = -0.0721, p = 0.0021).

Although DCSFormer does not achieve a statistically significant improvement over Point Transformer V3, it is worth noting that Point Transformer V3 itself does not show a significant gain over the original Point Transformer. In contrast, DCSFormer demonstrates a statistically significant improvement compared to Point Transformer (p = 0.0045), indicating that the modifications introduced in DCSFormer are effective. The lack of statistical significance relative to Point Transformer V3 may be due to the relatively small dataset, which leads to higher performance variability.

### 3.5 Visualize results comparison

To visually demonstrate the effectiveness of each model in the cotton seedling point cloud organ segmentation task and to compare their segmentation performance, representative cotton point cloud samples were selected from the test set for visualization. It should be noted that the completeness of pot point clouds varies across different samples, mainly due to objective factors during data acquisition and processing. However, since the primary focus of this study is on the geometric structure and semantic segmentation of plant organs, these variations do not materially affect the experimental results or conclusions.

**Figure 2:**
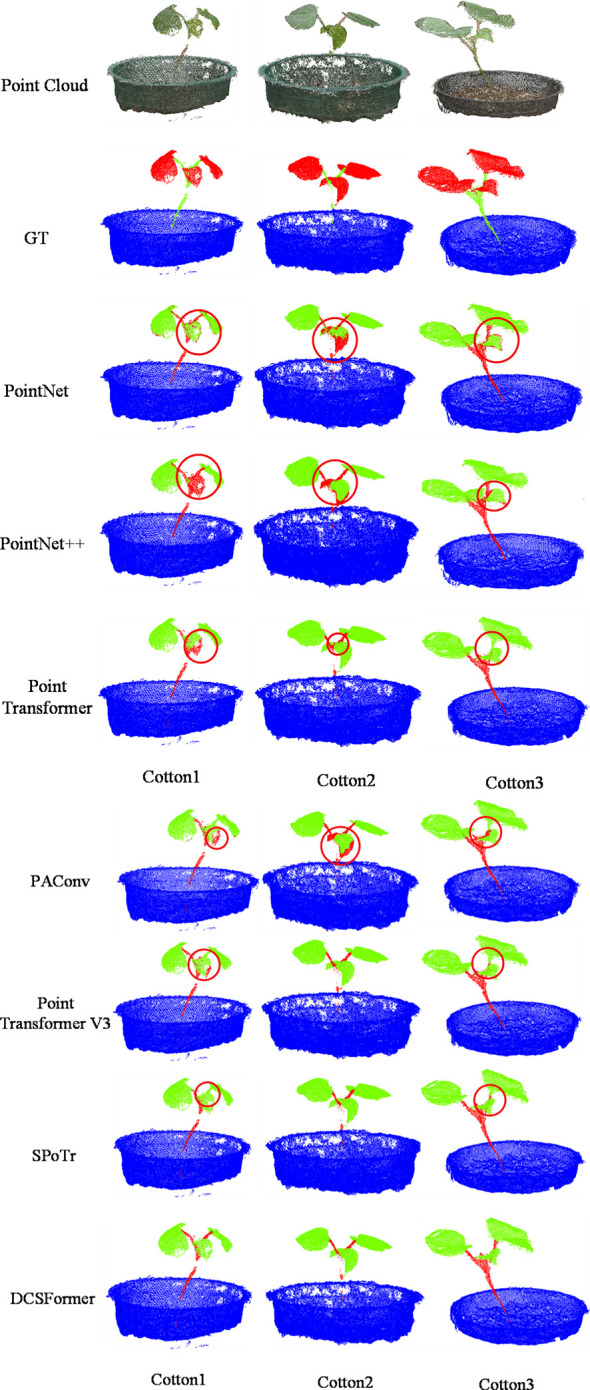
Qualitative comparison results on cotton datasets, illustrating several representative point cloud samples. From top to bottom: raw point cloud, ground truth (GT), segmentation results of each model. The regions highlighted with red circles indicate noticeable segmentation errors.

As shown in **Figure 2**, subjective analysis of the comparative results indicates that PointNet and PointNet++ generally exhibit mis-segmentation of leaves as stems. Specifically, in the segmentation results of cotton1 by PointNet++ and cotton2 by PointNet, this mis-segmentation is particularly pronounced, with large portions of leaf regions incorrectly identified as stems. In contrast, other comparison models do not show extensive mis-segmentation in leaf regions, but some errors remain at the junctions between stems and leaves. For example, in cotton1 and cotton3, nearly all comparative models exhibit local stem regions being missegmented as leaves.

By comparison, DCSFormer shows no obvious segmentation errors in the same samples. To provide a comprehensive view of the model, representative examples of its occasional segmentation errors, along with brief analyses, are included in the Supplementary Material. Overall, DCSFormer not only improves the accuracy of organ segmentation but also effectively captures stem-leaf boundaries and the overall geometric structure of the plants.

### 3.6 Performance on plant traits

To evaluate the biological relevance of the proposed DCSFormer, we applied the model’s segmentation outputs to measure plant height and canopy width using a minimum bounding rectangle approach. The predicted trait values were compared with manually measured ground-truth data, and the performance was quantified using the coefficient of determination (R²), root mean square error (RMSE), mean absolute percentage error (MAPE), and mean absolute error (MAE), as summarized in **Table 10**.

**Table 10:** Evaluation metrics for plant height and canopy width estimation.

Trait	R^2^	RMSE	MAPE (%)	MAE
Plant Height	0.678	0.513	2.474	0.371
Canopy Width	0.724	0.387	2.145	0.279

The results indicate that DCSFormer’s segmentation outputs effectively support the extraction of plant morphological traits, with R² values of 0.678 and 0.724, demonstrating a strong correlation with actual measurements. The low RMSE, MAE, and MAPE further confirm that the predicted trait values closely match the ground truth, with relative errors generally below 2.5%. Although small segmentation errors may affect trait estimates, for example, slightly under- or overestimating plant height or canopy width, the observed deviations are minor, indicating that DCSFormer’s segmentation quality is sufficient for reliable trait quantification.

These findings demonstrate the practical potential of DCSFormer for extracting biologically meaningful traits from 3D point clouds, thereby supporting downstream phenotyping and quantitative plant analysis. It should be noted that this analysis provides a preliminary validation focused on two key traits, and the framework can be extended in future work to measure additional morphological and physiological traits for more comprehensive plant phenotyping.

## 4 Discussion

### 4.1 Conceptual comparison

This study employs a cost-effective multi-view image approach to generate 3D plant point clouds. We then employ the improved PTv3-based semantic segmentation model, DCSFormer, to achieve high-precision segmentation of plant point clouds. Compared with organ segmentation networks such as CotSegNet and Panicle-3D, DCSFormer differs at the architectural level rather than merely in backbone selection.

The introduced mixture-of-experts (MoE) attention mechanism enables expert-specific feature extraction tailored to heterogeneous organ shapes, allowing the network to better handle thin, elongated stems and broad, overlapping leaves. Additionally, the proposed CLFSkip module performs frequency-aware cross-layer fusion, combining high-frequency geometric details from shallow layers with low-frequency structural information from deeper layers. Compared with conventional methods relying on purely spatial fusion or fixed attention patterns, this design facilitates the extraction of richer and more robust features.

### 4.2 Workflow scalability and automation feasibility

Although the current workflow still relies on manual selection of high-quality images, this step can be automated in high-throughput phenotyping settings. In our preliminary experiment, we evaluated eight commonly used blur-detection metrics—entropy, Brenner, Laplacian, SMD, SMD2, variance, energy, and Vollath (detailed results are provided in the Supplementary Material). Among these, the Laplacian-based method demonstrated the best overall performance in terms of accuracy and stability, indicating that this screening step can be effectively automated in practical applications. The subsequent SfM–MVS reconstruction is fully automated, and its scalability is primarily determined by the available computational resources.

To assess the practical feasibility of the entire pipeline, we measured the time required for each stage. Video acquisition takes approximately 12–15 seconds per plant; image extraction and manual screening require 1.5–2.0 minutes; point-cloud reconstruction takes 3–5 minutes; and point-cloud preprocessing requires about 20–30 seconds. When the automated blur-detection algorithm is used, the per-plant image screening time is reduced to 10–20 seconds, representing a substantial speed-up compared with fully manual filtering. Under automated screening, the total processing time per sample is approximately 4–6 minutes.

It should be noted that the automated blur-detection component represents preliminary work and has not yet undergone systematic evaluation. For this reason, and to ensure full control and reproducibility, the dataset released in this study is still based on manually screened images. Future work will focus on integrating a fully automated screening procedure and conducting comprehensive validation on large-scale datasets.

### 4.3 DCSFormer on different types of plant

To further investigate the generalization capability of the DCSFormer model in plant point cloud organ segmentation, two publicly available plant point cloud datasets, Pheno4D (Schunck et al., 2021) and Crops3D (Zhu et al., 2024), were selected. Transferability experiments were conducted to evaluate the segmentation performance of DCSFormer on other plant species, and comparisons were made with the baseline model. It should be noted that due to the large scale of these datasets and shared memory limitations on the current computing platform, test-time data augmentation was not applied. This adjustment may slightly reduce the final evaluation metrics, but the training hyperparameters and training intensity were kept consistent, ensuring fairness during the training process.

From the Pheno4D dataset, the Tomato class was selected, while from Crops3D, the Potato and Rapeseed classes were chosen. These three crops were selected because they exhibit distinct differences in leaf morphology, structural complexity, and point cloud characteristics: tomato plants have relatively broad leaves and branching structures; potato plants are relatively compact; and rapeseed has numerous densely distributed leaves. By including plant point clouds with such significant differences, the transferability of the model for stem and leaf segmentation across diverse crop morphologies can be more comprehensively evaluated. The selection of these two datasets thus provides a solid foundation for assessing the generalization ability of the model.

**Table 11** presents the per-class mIoU values of the three crops in the two public datasets for DCSFormer and its baseline model. Analysis of the experimental results across different crop datasets highlights the advantages and applicability of DCSFormer. For potato and tomato, which have relatively clear branching and well-defined structural hierarchies, DCSFormer improves mIoU by 3.15% and 2.79%, respectively, compared to the baseline. This indicates that the model is better at capturing fine-grained structures and small regions often overlooked by the baseline.

**Table 11:** Comparison of Point Transformer V3 with DCSFormer on Crops3D and Pheno4D datasets.

Dataset	Species	Model	mIoU(%)	mPrec(%)	mRec(%)	mF1(%)
Crops3D	Potato	Point Transformer V3	75.13	**88.25**	81.36	84.36
		DCSFormer	**78.28**	87.66	**85.83**	**86.71**
	Rapeseed	Point Transformer V3	71.45	77.84	79.69	78.71
		DCSFormer	74.19	82.21	80.61	81.28
Pheno4D	Tomato	Point Transformer V3	90.00	94.64	94.29	94.46
		DCSFormer	**92.79**	**97.74**	**94.49**	**96.05**

Bold type indicates the best result achieved in the corresponding indicator.

For rapeseed, a small-leaf, densely structured crop, DCSFormer achieves a mIoU increase of 2.74% accompanied by a Precision improvement of 4.37%, indicating that it effectively reduces misclassification and enhances class discriminability. In the tomato subset of Pheno4D, although the baseline already demonstrates strong performance, DCSFormer still achieves a notable improvement (+2.79% mIoU), with Precision increasing from 94.64% to 97.74%. Even on high-quality datasets with strong baselines, DCSFormer further optimizes segmentation accuracy and reduces false positives. Overall, DCSFormer outperforms the baseline across all metrics for different crop types, demonstrating stronger generalization in geometric detail modeling, cross-organ feature discrimination, and background suppression.

It should be noted that while quantitative evaluation metrics effectively assess segmentation accuracy across crops and organs, they do not fully reveal the model’s potential advantages in fine-grained structure modeling and boundary distinction. To address this, qualitative visual analyses were performed to compare the segmentation results of the baseline and DCSFormer, further validating the model’s performance in complex plant point cloud scenarios.

**Figure 3:**
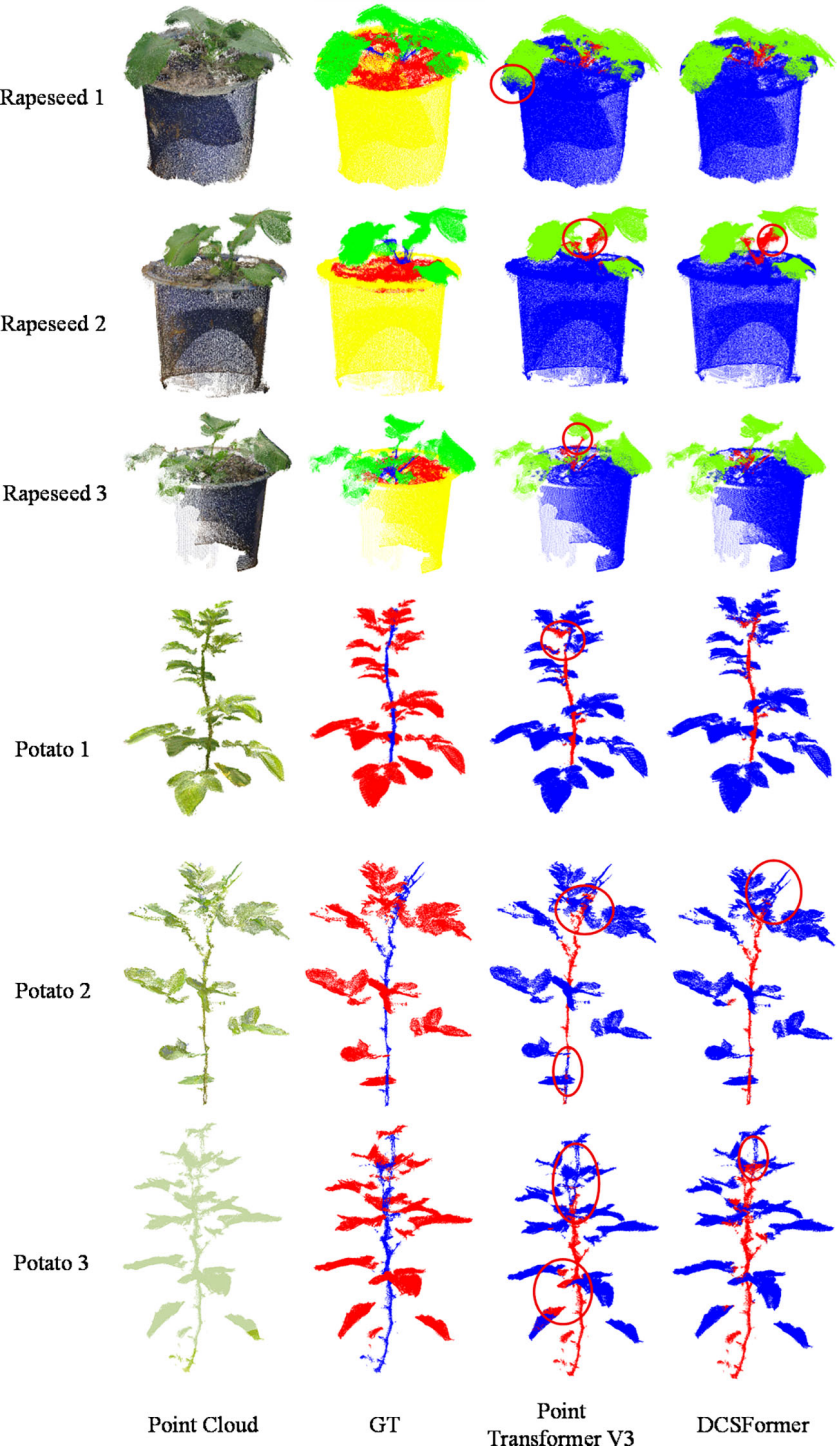
Qualitative comparison results on Crops3D datasets, illustrating several representative point cloud samples. From left to right: raw point cloud, ground truth (GT), segmentation result of Point Transformer, and segmentation result of DCSFormer. The regions highlighted with red circles indicate noticeable segmentation errors.

In **Figure 3**, qualitative comparisons on the Crops3D dataset are shown. For rapeseed, both models segment non-plant regions accurately, indicating these features are relatively easy to distinguish. However, in the main plant body, the baseline exhibits noticeable mis-segmentation at most stem-leaf junctions, resulting in blurred boundaries. In contrast, DCSFormer excels at recognizing such fine-grained structures, maintaining stem-leaf integrity and accurately distinguishing connected regions.

For potato, the baseline’s shortcomings are more pronounced. For instance, in the potato2 sample, the baseline incorrectly segments parts of the stem as leaves, while in potato1 and potato3, some leaves are misclassified as stems. This instability indicates limitations in handling complex geometries and subtle morphological differences. DCSFormer, by contrast, achieves accurate stem-leaf segmentation in most cases, with only minor mis-segmentation in high-density local point cloud regions of potato1 and potato2, demonstrating overall superiority over the baseline.

**Figure 4:**
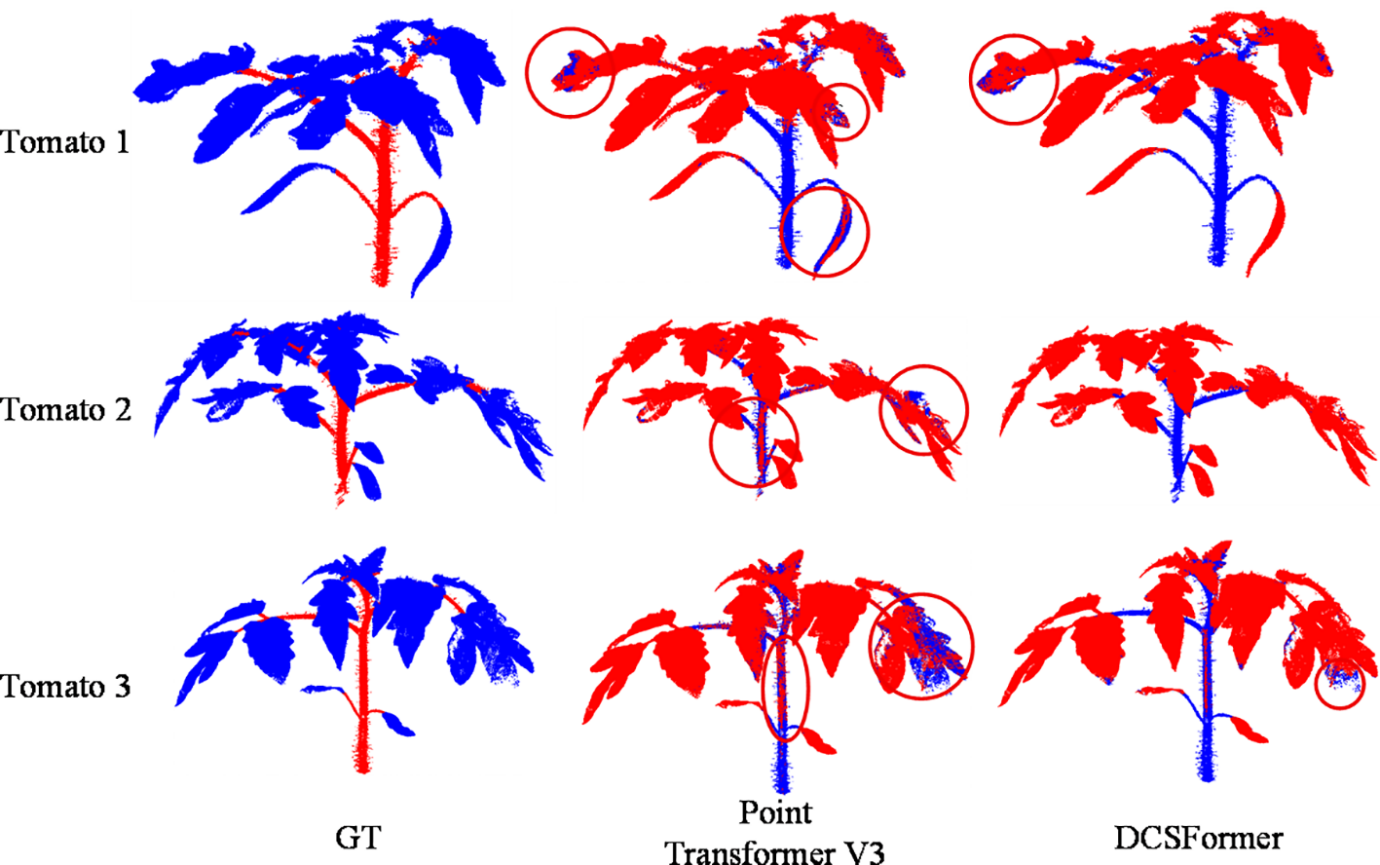
Qualitative comparison results on Pheno4D datasets, illustrating several representative point cloud samples. From left to right: ground truth (GT), segmentation result of Point Transformer, and segmentation result of DCSFormer. The regions highlighted with red circles indicate noticeable segmentation errors.

**Figure 4** shows the qualitative comparison for the tomato class in Pheno4D. The baseline model exhibits prominent mis-segmentation across multiple samples. Specifically, in all three tomato samples, leaves are frequently misclassified as stems; additionally, in tomato2 and tomato3, some leaf regions are misclassified in stem areas. These errors not only reduce overall segmentation accuracy but are particularly evident in structurally complex organ regions. By contrast, DCSFormer achieves more precise segmentation in the same scenarios, with only a few leaves misclassified as stems in tomato1 and tomato3, and minimal leaf contamination in the stem of tomato2. Overall, DCSFormer better preserves organ class consistency.

Furthermore, the demonstrated generalization of DCSFormer across Pheno4D and Crops3D datasets suggests its potential integration into high-throughput phenotyping pipelines, where large-scale 3D plant scans could be processed efficiently. Future work could also explore leveraging multi-modal data, such as RGB, hyperspectral, or LiDAR inputs, to further enhance segmentation performance and trait extraction, broadening the applicability of DCSFormer to diverse phenotyping scenarios.

## 5 Conclusions

In the cotton seedling point cloud organ segmentation task, DCSFormer demonstrates outstanding performance. During feature extraction, the model incorporates the DCS Block, which employs multi-expert sparse routing and a dual-channel attention mechanism to effectively balance local geometric details and global semantic dependencies, enhancing the model’s ability to distinguish leaf boundaries and slender stems. In the feature fusion stage, the CLFSkip module replaces the traditional skip connection, using a multi-dimensional feature extractor and cross-layer fusion mechanism to integrate shallow geometric features with deep semantic features. This design overcomes the limitations of conventional skip connections in information propagation and feature interaction, enabling fine-grained boundary reconstruction while maintaining overall semantic consistency.

Experimental results show that DCSFormer outperforms advanced comparison models across all four metrics—mIoU, mPrec, mRec, and mF1—achieving a maximum of 93.67% mIoU and 96.56% mF1, validating the effectiveness of the proposed approach. Furthermore, in cross-crop transfer experiments, DCSFormer achieves significant improvements over the baseline on tomato, potato, and rapeseed point cloud datasets, demonstrating strong generalization ability and adaptability.

Despite its superior performance, DCSFormer’s Transformer-based architecture entails relatively high computational cost, posing challenges for real-time or resource-constrained applications. Additionally, this study focuses on a single cultivar (“Xinjiang Big Peach Cotton”) collected under a controlled acquisition setup and based on a relatively small dataset. The dataset does not include samples with leaf or stem damage caused by pests, disease, or mechanical stress. These factors may introduce a potential domain gap when transferring the model to field conditions or to more complex real-world scenarios. Therefore, the model’s generalizability to other cultivars, growth stages, and more complex outdoor conditions requires further validation.

Future work will proceed in several directions: first, introducing model compression and lightweight design to reduce parameter size and inference latency, improving practical applicability; second, expanding the application to point cloud organ segmentation for diverse crops to further explore model generality, as well as extending the framework toward characterizing the developmental progression of individual plants over time; and finally, integrating instance segmentation and morphological feature extraction techniques to advance high-throughput phenotyping research and intelligent agricultural applications.

## Data Availability

The datasets presented in this study can be found in online repositories. The names of the repository/repositories and accession number(s) can be found below: https://www.kaggle.com/datasets/tengfeiliu333/dcsformer-cotton/croissant/download.

## References

[B1] Chu P. Han B. Guo Q. Wan Y. Zhang J. (2025). A three-dimensional phenotype extraction method based on point cloud segmentation for all-period cotton multiple organs. Plants 14, 1578. doi: 10.3390/plants14111578, PMID: 40508253 PMC12157811

[B2] Elman J. L. (1990). Finding structure in time. Cogn. Sci. 14, 179–211. doi: 10.1207/s15516709cog1402_1

[B3] Gong L. Du X. Zhu K. Lin K. Lou Q. Yuan Z. . (2021). Panicle-3D: Efficient Phenotyping Tool for Precise Semantic Segmentation of Rice Panicle Point Cloud. Plant Phenomics. 2021, 9838929. doi: 10.34133/2021/9838929, PMID: 35024618 PMC8720256

[B4] Gu Y. Zhong Z. Wu S. Xu Y. (2017). “ Enlarging effective receptive field of convolutional neural networks for better semantic segmentation,” in 2017 4th IAPR Asian Conference on Pattern Recognition (ACPR) (Nanjing, China: IEEE). 388–393. doi: 10.1109/ACPR.2017.7

[B5] Guo Y. Wang H. Hu Q. Liu H. Liu L. Bennamoun M. (2020). Deep learning for 3d point clouds: A survey. IEEE Trans. Pattern Anal. Mach. Intell. 43, 4338–4364. doi: 10.1109/TPAMI.2020.3005434, PMID: 32750799

[B6] Hu J. Shen L. Sun G. (2018). Squeeze-and-excitation networks. Proc. IEEE Conf. Comput. Vision Pattern recognition, 7132–7141. doi: 10.48550/arXiv.1709.01507

[B7] Jaderberg M. Simonyan K. Zisserman A. (2015). Spatial transformer networks. Adv. Neural Inf. Process. Syst. 28. doi: 10.5555/2969442.2969465

[B8] Jiang L. Li C. Fu L. (2025). Apple tree architectural trait phenotyping with organ-level instance segmentation from point cloud. Comput. Electron. Agric. 229, 109708. doi: 10.1016/j.compag.2024.109708

[B9] Johri P. Kim S. Dixit K. Sharma P. Kakkar B. Kumar Y. . (2024). Advanced deep transfer learning techniques for efficient detection of cotton plant diseases. Front. Plant Sci. 15. doi: 10.3389/fpls.2024.1441117, PMID: 39759238 PMC11696538

[B10] Kim Y. (2014). Convolutional neural networks for sentence classification. arXiv preprint. doi: 10.48550/arXiv.1408.5882

[B11] Lei L. Yang Q. Yang L. Shen T. Wang R. Fu C. (2024). Deep learning implementation of image segmentation in agricultural applications: a comprehensive review. Artif. Intell. Rev. 57, 149. doi: 10.1007/s10462-024-10775-6

[B12] Miao T. Zhu C. Xu T. Yang T. Li N. Zhou Y. . (2021). Automatic stem-leaf segmentation of maize shoots using three-dimensional point cloud. Comput. Electron. Agric. 187, 106310. doi: 10.1016/j.compag.2021.106310

[B13] Minervini M. Scharr H. Tsaftaris S. A. (2015). Image analysis: the new bottleneck in plant phenotyping [applications corner]. IEEE signal processing magazine. 32, 126–131. doi: 10.1109/MSP.2015.2405111

[B14] Muraleedharan V. Rajan S. C. (2024). Geometric entropy of plant leaves: A measure of morphological complexity. PloS One 19, e0293596. doi: 10.1371/journal.pone.0293596, PMID: 38166118 PMC10760904

[B15] Park J. Lee S. Kim S. Xiong Y. Kim H. J. (2023). “ Self-positioning point-based transformer for point cloud understanding,” in IEEE/CVF conference on computer vision and pattern recognition (Vancouver, Canada: IEEE). 21814–21823. doi: 10.48550/arXiv.2303.16450

[B16] Qi C. R. Su H. Mo K. Guibas L. J. (2017b). “ Pointnet: Deep learning on point sets for 3d classification and segmentation,” in IEEE Conference on Computer Vision and Pattern Recognition (Honolulu, United States: IEEE). 652–660. doi: 10.48550/arXiv.1612.00593

[B17] Qi C. R. Yi L. Su H. Guibas L. J. (2017a). Pointnet++: Deep hierarchical feature learning on point sets in a metric space. Adv. Neural Inf. Process. Syst. 30. doi: 10.48550/arXiv.1706.02413

[B18] Rehman A. Farooq M. (2019). Morphology, physiology and ecology of cotton. Cotton production, 23–46. doi: 10.1002/9781119385523.ch2

[B19] Ronneberger O. Fischer P. Brox T. (2015). “ U-net: Convolutional networks for biomedical image segmentation,” in International Conference on Medical image computing and computer-assisted intervention ( Springer Verlag). 234–241. doi: 10.1007/978-3-319-24574-4_28

[B20] Scarselli F. Gori M. Tsoi A. C. Hagenbuchner M. Monfardini G. (2008). The graph neural network model. IEEE Trans. Neural Networks 20, 61–80. doi: 10.1109/TNN.2008.2005605, PMID: 19068426

[B21] Schunck D. Magistri F. Rosu R. A. Cornelißen A. Chebrolu N. Paulus S. . (2021). Pheno4D: A spatio-temporal dataset of maize and tomato plant point clouds for phenotyping and advanced plant analysis. PloS One 16, e0256340. doi: 10.1371/journal.pone.0256340, PMID: 34407122 PMC8372960

[B22] Song J. Ma B. Xu Y. Yu G. Xiong Y. (2025). Organ segmentation and phenotypic information extraction of cotton point clouds based on the CotSegNet network and machine learning. Comput. Electron. Agric. 236, 110466. doi: 10.1016/j.compag.2025.110466

[B23] Tietze H. Abdelhakim L. Pleskačová B. Kurtz-Sohn A. Fridman E. Nikoloski Z. . (2025). Prediction of harvest-related traits in barley using high-throughput phenotyping data and machine learning. Front. Plant Sci. 16. doi: 10.3389/fpls.2025.1686506, PMID: 41164241 PMC12560056

[B24] Vaswani A. Shazeer N. Parmar N. Uszkoreit J. Jones L. Gomez A. N. . (2017). Attention is all you need. Adv. Neural Inf. Process. Syst. 30. doi: 10.48550/arXiv.1706.03762

[B25] Wang Y. Sun Y. Liu Z. Sarma S. E. Bronstein M. M. Solomon J. M. (2019). Dynamic graph cnn for learning on point clouds. ACM Trans. Graph. 38, 1–12. doi: 10.1145/3326362

[B26] Wu X. Jiang L. Wang P. S. Liu Z. Liu X. Qiao Y. . (2024). “ Point transformer v3: Simpler faster stronger,” in IEEE/CVF conference on computer vision and pattern recognition (Seattle, United States: IEEE). 4840–4851. doi: 10.48550/arXiv.2312.10035

[B27] Xu M. Ding R. Zhao H. Qi X. (2021). “ Paconv: Position adaptive convolution with dynamic kernel assembling on point clouds,” in IEEE/CVF conference on computer vision and pattern recognition (Nashville, United States: IEEE). 3173–3182. doi: 10.48550/arXiv.2103.14635

[B28] Ye D. Wu L. Li X. Atoba T. O. Wu W. Weng H. (2023). A synthetic review of various dimensions of non-destructive plant stress phenotyping. Plants 12, 1698. doi: 10.3390/plants12081698, PMID: 37111921 PMC10146287

[B29] Zhang J. Zhao X. Chen Z. Lu Z. (2019). A review of deep learning-based semantic segmentation for point cloud. IEEE Access 7, 179118–179133. doi: 10.1109/ACCESS.2019.2958671

[B30] Zhao C. Zhang Y. Du J. Guo X. Wen W. Gu S. . (2019). Crop phenomics: current status and perspectives. Front. Plant Sci. 10. doi: 10.3389/fpls.2019.00714, PMID: 31214228 PMC6557228

[B31] Zhao H. Jiang L. Jia J. Torr P. H. Koltun V. (2021). “ Point transformer,” in IEEE/CVF international conference on computer vision (Montreal, Canada). 16259–16268. doi: 10.48550/arXiv.2012.09164

[B32] Zhao S. Huang G. Yang S. Wang C. Wang J. Zhao Y. . (2025). Precise 3D geometric phenotyping and phenotype interaction network construction of maize kernels. Front. Plant Sci. 16. doi: 10.3389/fpls.2025.1438594, PMID: 40265111 PMC12011857

[B33] Zhou J. Fu X. Zhou S. Zhou J. Ye H. Nguyen H. T. (2019). Automated segmentation of soybean plants from 3D point cloud using machine learning. Comput. Electron. Agric. 162, 143–153. doi: 10.1016/j.compag.2019.04.014

[B34] Zhu J. Zhai R. Ren H. Xie K. Du A. He X. . (2024). Crops3D: a diverse 3D crop dataset for realistic perception and segmentation toward agricultural applications. Sci. Data 11, 1438. doi: 10.1038/s41597-024-04290-0, PMID: 39730336 PMC11681092

