# Supplementary material for: DCSFormer: a high-precision method for cotton seedling point cloud organ segmentation
Source: Front Plant Sci. 2026 Jan 22;16:1724451. doi: 10.3389/fpls.2025.1724451 (PMC12872859; doi:10.3389/fpls.2025.1724451)
Supplement: Supplementary file 1 [file DataSheet1.pdf]

Supplementary Material

1 Supplementary Figures and Tables

1.1 Supplementary Tables S1

Supplementary Table S1 The specific training hyperparameters used for each model

Model	Batch Size	Learning Rate	Optimizer	Epochs	LR Scheduler / Decay
PointNet	32	0.001	Adam	250	Step decay: step=200000, γ=0.7
PointNet++	16	0.001	Adam	250	Step decay: step=200000, γ=0.7
Point Transformer	2	0.006	AdamW	3000	MultiStepLR, milestones=[0.6,0.8], γ=0.1
PAConv	8	0.2	SGD	150	CosineAnnealingLR, eta_min=0.002, T_max=150
Point Transformer V3	2	0.002	AdamW	3000	OneCycleLR, max_lr=[0.002,0.0002], pct_start=0.05, anneal_strategy=cos
SPoTr	32	0.01	AdamW	100	Cosine scheduler, min_lr=1e-5
DCSFormer	2	0.002	AdamW	3000	OneCycleLR, max_lr=[0.002,0.0002], pct_start=0.05, anneal_strategy=cos

1.2 Supplementary Figures 1

A preliminary experiment was conducted using the same set of collected images for consistency. Within this dataset, manual inspection identified two images as blurred. Eight commonly used blur-detection metrics—entropy, Brenner, Laplacian, SMD, SMD2, variance, energy, and Vollath—were evaluated on this dataset. The detailed results of this comparative analysis are provided in Supplementary Figure 1.

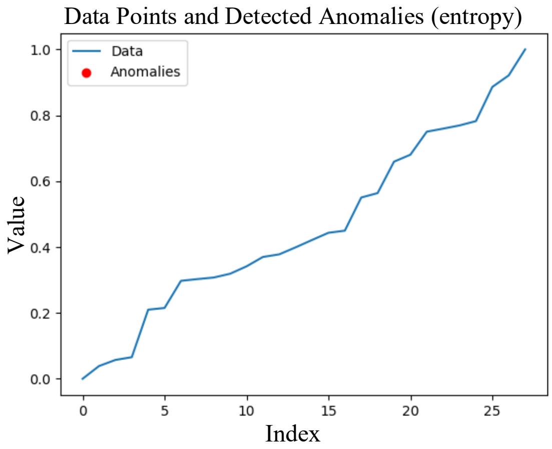
Data Points and Detected Anomalies (entropy)
Data
Anomalies
Value
Index
0.0
0.2
0.4
0.6
0.8
1.0
0
5
10
15
20
25

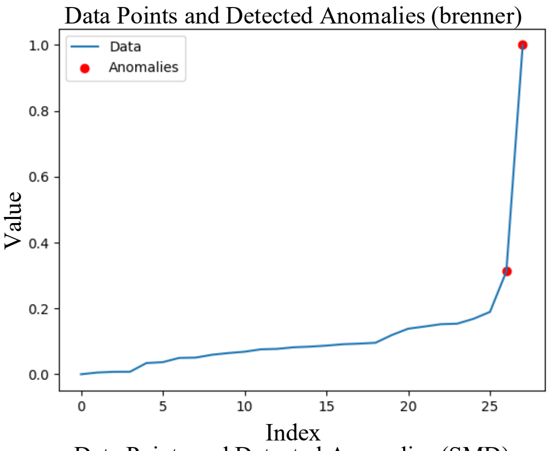
Data Points and Detected Anomalies (brenner)
Data
Anomalies
Value
Index
0.0
0.2
0.4
0.6
0.8
1.0
0
5
10
15
20
25

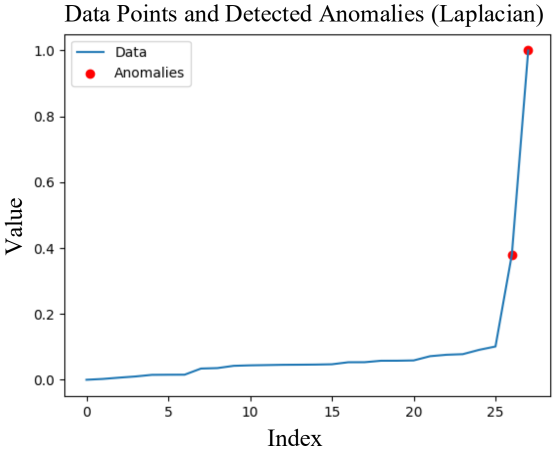
Data Points and Detected Anomalies (Laplacian)
Data
Anomalies
Value
Index
0.0
0.2
0.4
0.6
0.8
1.0
0
5
10
15
20
25

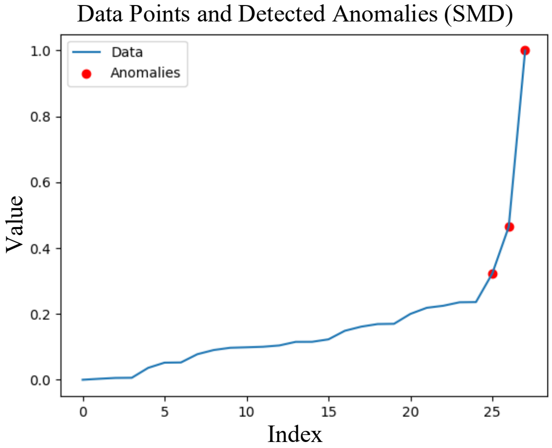
Data Points and Detected Anomalies (SMD)
Data
Anomalies
Value
Index
0.0
0.2
0.4
0.6
0.8
1.0
0
5
10
15
20
25

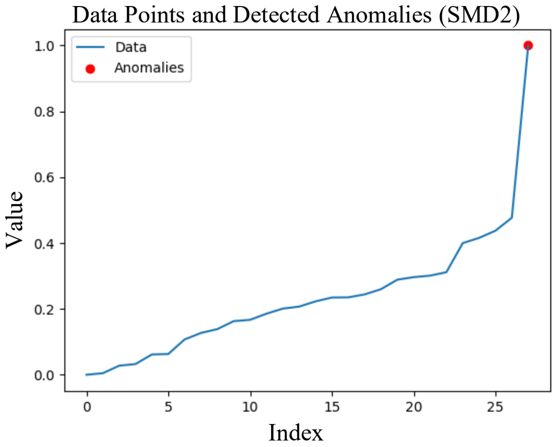
Data Points and Detected Anomalies (SMD2)
Data
Anomalies
Value
Index
0.0
0.2
0.4
0.6
0.8
1.0
0
5
10
15
20
25

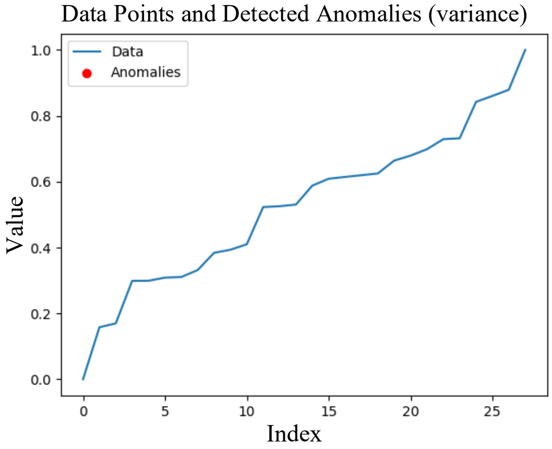
Data Points and Detected Anomalies (variance)
Data
Anomalies
Value
Index
0.0
0.2
0.4
0.6
0.8
1.0
0
5
10
15
20
25

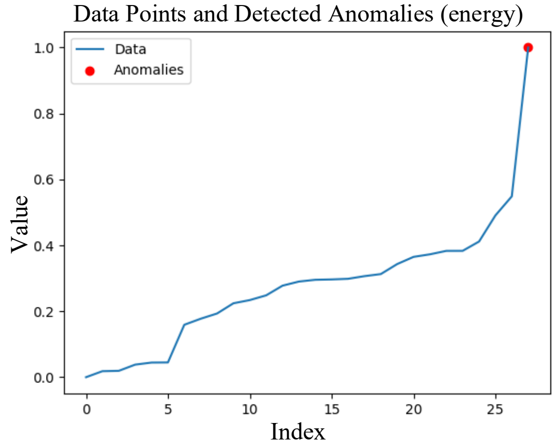
Data Points and Detected Anomalies (energy)
Data
Anomalies
Value
Index
0.0
0.2
0.4
0.6
0.8
1.0
0
5
10
15
20
25

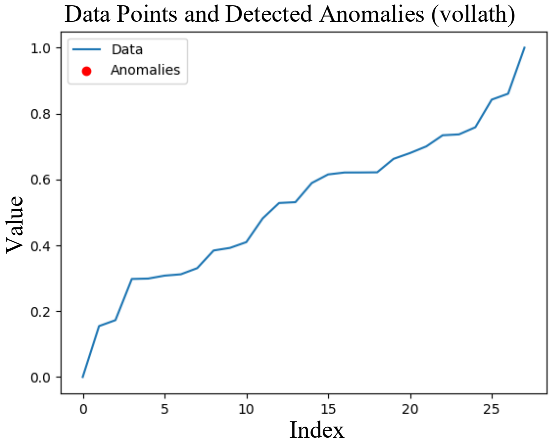
Data Points and Detected Anomalies (vollath)
Data
Anomalies
Value
Index
0.0
0.2
0.4
0.6
0.8
1.0
0
5
10
15
20
25

Supplementary Figure 1. Comparison of eight blur-detection metrics used for automated image quality screening. Red dots indicate the images flagged as blurred by each corresponding metric, illustrating their differences in sensitivity and detection behavior.

1.3 Supplementary Figures 2

Although DCSFormer achieves high accuracy in cotton seedling point cloud segmentation, occasional errors were observed in a few challenging cases. Representative examples are shown in Supplementary Figure 2. Most errors occur at the boundaries between two organs, such as cotyledons or leaf-stem junctions, where points are sparse and local features are ambiguous, leading to occasional misclassification. Despite these minor errors, the overall segmentation quality remains high, as reflected in the quantitative metrics presented in the main text. Most errors are localized and have minimal impact on downstream phenotypic trait extraction, such as plant height or canopy width.

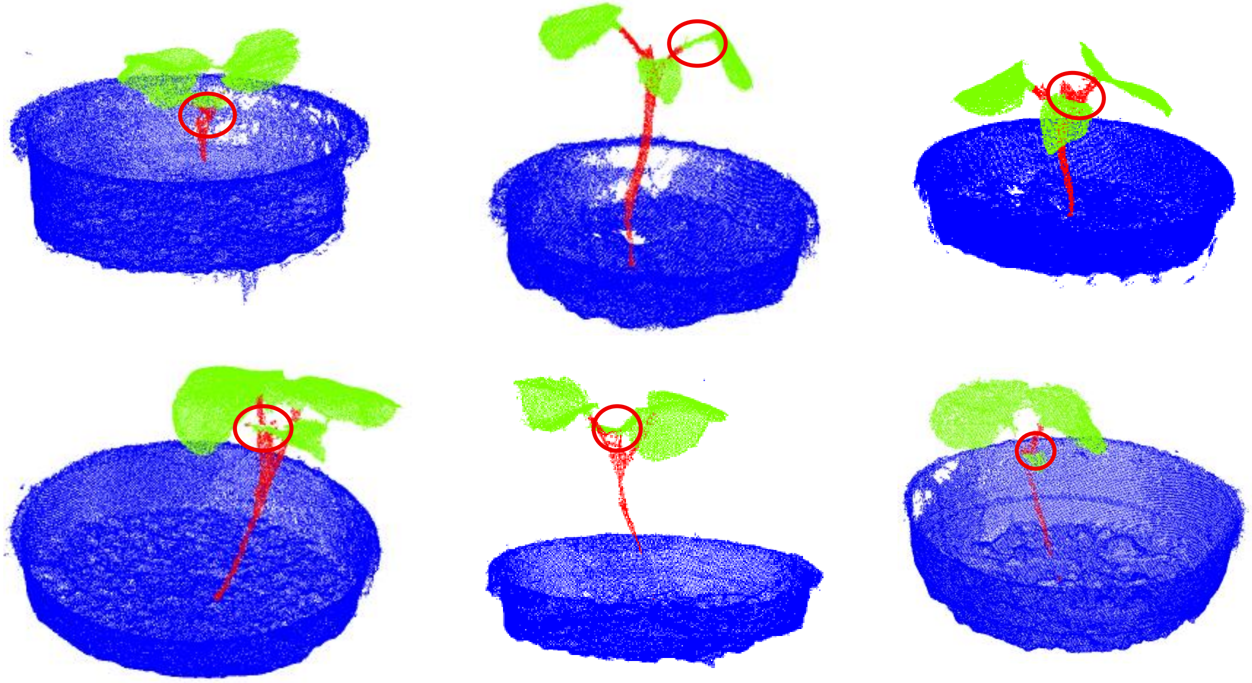

Supplementary Figure 2. Examples of segmentation errors produced by DCSFormer on cotton seedling point clouds.
